# Herding or wisdom of the crowd? Controlling efficiency in a partially rational financial market

**DOI:** 10.1371/journal.pone.0239132

**Published:** 2020-09-11

**Authors:** Fabio Della Rossa, Lorenzo Giannini, Pietro DeLellis

**Affiliations:** 1 Department of Electronic, Information, and Bioengineering, Politecnico di Milano, Milan, Italy; 2 Department of Electrical Engineering and Information Technology, University of Naples Federico II, Naples, Italy; Unviersity of Burgundy, FRANCE

## Abstract

Herding has often been blamed as one of the possible causes of market instabilities, ultimately yielding to bubbles and crushes. On the other hand, researchers hypothesized that financial systems may benefit from the so-called *wisdom of the crowd*. To solve this apparent dichotomy, we leverage a novel financial market model, where the agents form their expectations by combining their individual return estimation with the expectations of their neighbors. By establishing a link between herding, sociality, and market instabilities, we point out that the emergence of collective decisions in the market is not necessarily detrimental. Indeed, when all the agents tend to conform their expectations to those of one or few leaders, herding might dramatically reduce market efficiency. However, when each agent accounts for a plurality of opinions, thus following the wisdom of the crowd, market dynamics become efficient. Following these observations, we propose two alternative control strategies to reduce market instability and enhance its efficiency.

## Introduction

In neoclassical economics, the efficient market assumption prescribes that the market price of an asset fully reflects all available information [1]. This implies that financial agents form rational expectations about future price variations, thus driving the market price towards the correct *fundamental value* of the traded asset [2]. This theory has been severely criticized in the last decades, since it failed to explain periods when the market consistently misestimates the value of an asset [3]. A possible explanation of this empirical evidence is that the decision-making in humans is not perfectly rational, and might be subservient to emotions and cognitive biases [4, 5]. Also, individual decisions are not taken independent of the opinions and behaviors of the other investors, see for instance the behavior of trend followers, which might contribute to trigger market instabilities [6]. Several financial bubbles are induced by similar mechanisms, as in the so-called *dot-com boom* that took place at the beginning of the 21st century, where investors overlooked traditional metrics of profitability [7], leading to a rapid increase and subsequent decrease of the share prices of the IT companies.

Considering their potentially disruptive socio-economic impact, identifying the mechanisms underlying the emergence of *market bubbles* is paramount [8, 9]. Since market crashes are observed in the presence of a significant difference between the market prices and the real value of the traded assets, their emergence is not compatible with the traditional efficient market assumption. A crucial step towards understanding the market dynamics has been made by behavioral economics, which contributed a better characterization of the human decision process, pervaded by irrationality and uncertainties [10]. This yielded a more accurate atomized characterization of the human behavior that, paired with the increased computational power, allowed for the development of *artificial financial markets* trying to reproduce in a simulated environment the features of real financial markets [11]. A considerable research effort has been devoted to decipher the effect of imitative mechanisms in financial markets, see e.g. [12–14]. Indeed, the financial agents, not being aware of the of the intrinsic value of a resource, may leverage the opinion of her peers to form their expectation. In the last decade, the presence of imitative behaviors in trading has been magnified by social trading platforms like *eToro* and *Zulutrade* [15], which allow investors to observe the trading patterns of their peers and copy their investment strategies.

In the literature, there is no consensus on the impact of imitation on the market dynamics. Often, when the emergence of a collective decision in the market is identified as one of the possible causes of market instabilities, the derogatory word *herding* is used [16, 17]. The use of this term to indicate the tendency to (blindly) imitate the behavior of other individuals is rather consistent in the literature [18] and, interestingly, has a negative connotation also in other research fields, including in the studies on crowd formation during emergencies [19, 20]. In financial markets, herding has been viewed as a cascading imitation process that reinforces agent expectations [21], thus causing an increment in volatility [22]. On the other hand, considering the inherent uncertainties of financial markets, imitating the behavior of the average investor, with the agents following the so-called *wisdom of the crowd*, an ensemble of diversely informed individuals may outperform experts, and contribute to steer the asset price towards its fundamental value, thus enhancing market efficiency. Examples of this phenomenon can be found in prediction markets [23], see e.g. [24, 25]. A first attempt to discriminate between a detrimental herding and a *rational adaptation* has been made in [26], where the complexity of the agent individual behavior was neglected to focus instead on the dynamic evolution of the network structure.

This work leverages the tools of complex network theory to explain the apparent dichotomy between herding and crowd wisdom. In particular, we employ a recent financial market model [27] to elucidate the relationship between sociality, imitation, and market efficiency. Specifically, we consider a centralized market with a double auction order book, and model the social interactions among the agents through a graph. The agents do not possess a perfect knowledge of the current fundamental price, but they form their own prediction, which is then adjusted based on the predictions of the neighboring agents. By varying the degree of sociality among the agents, we observe that, when all the traders conform their expectations to those of a small subset of investors, then herding is associated to a decrease of market efficiency. On the contrary, in the absence of a dominant opinion in the market, its efficiency may benefit from the social interaction among the investors. Driven by these numerical observations, we devised two alternative control strategy, which can be readily translated into market policies in social trading platforms, where the interaction topology among the agents can be manipulated. The first control strategy consists in isolating (i.e. disconnecting) the most influential (i.e. the wealthiest) traders by not disclosing their expectations, while the second is a *data anonymization* strategy, where the expectations are disclosed to neighboring agents, but not their identities. A thorough statistical analysis illustrates how both strategies are effective in enhancing market efficiency even in the presence of a strong social influence among the investors. Indeed, we observe an instance of crowd wisdom, as the trading patterns become strongly correlated, but the market price tend to closely follow the fundamental price, this reflecting in a healthy and efficient market.

## Methods

### Double auction markets

In double auction markets [28], a centralized entity, i.e., the market institution, collects all the bids entered by the buyers and the offers submitted by the sellers, and store them in an *order book*. The bids (offers) of the traders consist in their willingness to buy (sell) a specific amount of goods at a *limit price*, which is the highest (lowest) price they are willing to accept for the trade. The order is stored in the book until another trader is willing to match the same price request, and it is *executed*. An order that is still not executed is called *limit order*. When a trader is willing to buy (sell) at a price another agent has already submitted a sell (buy) order, then her order is called *market order*, and is immediately executed. The highest of all the buy limit orders is the *bid price* *p*_bid_, while the lowest sell limit order is the *ask price* *p*_ask_. Market orders determine the execution of limit orders with limit price equal to the current bid and ask prices. The price of the last transaction is the current asset price *p*_cur_, while the asset price at the beginning of the *k*-th day of trading is called the *market price* *p*(*k*).

### Artificial financial market model

Here, we describe in details a recent agent-based model of a single-asset double auction market, which was first presented in [27]. The market is populated by a set of *N* financial agents, who submit market or limit orders depending on their expectation about the future prices of the asset. The wealth *W*_*i*_(*k*) of agent *i* at day *k* depends on the quantity *a*_*i*_(*k*) of the asset that she possesses, and her current cash availability *c*_*i*_(*k*). Indeed,

$$\begin{array}{c} W_{i}\left(k\right)=c_{i}\left(k\right)+a_{i}\left(k\right)p\left(k\right), \end{array}$$ (1)

for *i* = 1, …, *N*, $k\in Z^{+}$. Note that, when evaluating the wealth of an agent, we neglect price impact (i.e. the correlation between an incoming order and the subsequent price change [29]), and only consider the market price *p*(*k*) at the beginning of the *k*-th trading day.

A key feature of this model is that it explicitly accounts for the social interaction among the agents. As in social trading platforms, our market model assumes that traders are aware of the behavior of a subset of their peers. Specifically, the agents are supposed to interact on a directed graph G=(V,E), where V identifies the set of financial agents, while the presence of an edge (i,j)∈E implies that agent *i* has an influence over agent *j*. Indeed, when forming her expectations, the *i*-th agent only takes into account the decisions of the agents in her *in-neighborhood*

$$N_{i}^{\text{in}}=\left\{i\in V\;:\;\left(j,i\right)\in E\right\}.$$

Every day, the sequence of trading is randomly extracted, and each agent performs a prediction of the future returns and then decides to submit a market or limit order in the book.

#### Return prediction

At the *k*-th day of trading, the *i*-th agent makes a prediction ${\hat{r}}_{i}\left(k\right)$ of the expected log-return of the traded asset, obtained as a convex combination of the agent’s individual prediction ${\hat{r}}_{f,i}\left(t\right)$ and a social prediction ${\hat{r}}_{s,i}\left(t\right)$, that is a weighted average of the predictions of her in-neighbors.

*Individual prediction*. Different from the other market variables, the price prediction performed by each agent continuously updates with time. Namely, the dynamics of the prediction ${\hat{p}}_{f,i}$ of the fundamental price *p*_*f*_ performed by the *i*-th agent is modelled by the following Ornstein-Uhlenbeck process:

$$\begin{array}{c} \partial {\hat{p}}_{f,i}\left(t\right)=\gamma_{i}\left(p_{f}\left(t\right)-{\hat{p}}_{f,i}\left(t\right)\right)\partial t+\sigma_{i}\partial B_{i}\left(t\right), \end{array}$$ (2)

where $t\in R^{+}$, $\gamma_{i}\left(p_{f}\left(t\right)-{\hat{p}}_{f,i}\left(t\right)\right)\partial t$ is a mean reversion term that captures the tendency towards a correct estimation of the fundamental price, $\partial B_{i}\left(t\right)∼N\left(0,1\right)$ is a Wiener process modeling the inherent uncertainty affecting the estimation; the parameter *γ*_*i*_ determines the intensity of attraction towards *p*_*f*_, which can be considered as a measure of the agent *expertise*, that is, her ability in estimating the fundamental price. On the other hand, the higher is the *insecurity* of the agent, the higher the variance $\sigma_{i}^{2}$ of the Wiener process will be.

The goal of model (2) is to capture the tradeoff between the tendency of a financial agent i) to accurately identifying the fundamental price (this is captured by the mean reverting term of Eq (2)) and ii) to deviate from it (captured by the Wiener process). The tradeoff between these two terms is regulated by the ratio between the agent expertise *γ*_*i*_ and her insecurity *σ*_*i*_. Note that, to simulate the artificial financial market, we then need to assume a given fundamental value *p*_*f*_(*t*). In agreement with past works [30, 31], we take it as a realization of a geometric Wiener process. However, this is not assumed to be known by the agent.

In our study, we assume that all the prices and their estimations are positive. This is enforced through our model implementation (see the subsection Numerical Setup), where we take the fundamental price *p*_*f*_(*t*) at any time instant *t* sufficiently large if compared with the variance of the Wiener process, such that in all our repetitions ${\hat{p}}_{f}\left(t\right)$ is also positive. Therefore, during the *k*-th day of trading, the expected log-return is then estimated as

$$\begin{array}{c} {\hat{r}}_{f,i}\left(k\right)=\text{log}(\frac{{\hat{p}}_{f,i}\left(k\right)}{p_{\text{cur}}^{i}\left(k\right)}), \end{array}$$ (3)

where $p_{\text{cur}}^{i}\left(k\right)$ is the asset price before agent *i* participates to the *k*-th trading session.

*Social prediction*. As in social trading, we assume that the agents, when investing, take into account the behavior of a subset of their peers. In our model, the behavior of an agent is captured by her expected log-return, which influences both price and demand determination. Therefore, if the set of her in-neighbors $N_{i}^{\text{in}}$ is non-empty, agent *i*, when deciding how to invest (i.e. computing her expected return), performs a weighted average of the expected log-returns of her in-neighbors, thus building her *social prediction* on the returns. The weights are selected so that the most successful traders, which in our model are identified as the wealthiest, are weighted the most. In real markets, different proxies of success can be considered. Namely,

$$\begin{array}{c} {\hat{r}}_{s,i}\left(k\right)=\frac{1}{W_{\text{in}}\left(k\right)}(\sum_{j\in N_{i}^{\text{in}}}W_{j}\left(k\right){\hat{r}}_{j}\left(k-1\right)), \end{array}$$ (4)

where *W*_in_(*k*) is the sum of the wealth of the in-neighbors of *i* at the beginning of day *k*. Note that, since we assumed *p*(*k*) > 0, Eq (1) ensures that *W*_*j*_(*k*) for all j∈V and $k\in Z_{\geq 0}$, in turn guaranteeing that Eq (4) is well-posed.

*Overall prediction*. Agent *i* then computes the future log-return of the traded asset as a convex combination of the individual and social predictions:

$$\begin{array}{c} {\hat{r}}_{i}\left(k\right)=\left(1-\beta_{i}\left(k\right)\right){\hat{r}}_{f,i}\left(k\right)+\beta_{i}\left(k\right){\hat{r}}_{s,i}\left(k\right), \end{array}$$ (5)

where *β*_*i*_(*k*) is zero when $N_{i}^{\text{in}}=Ø$, otherwise it is computed as

$$\beta_{i}\left(k\right)=\zeta \;\frac{W_{\text{in}}\left(k\right)}{W_{\text{in}}\left(k\right)+W_{i}\left(k\right)}.$$

The constant parameter *ζ* ∈ [0, 1] is the network *sociability*, which balances the relevance of ${\hat{r}}_{s,i}$ against the individual prediction of the return ${\hat{r}}_{f,i}$. The factor *W*_in_(*k*)/(*W*_in_(*k*) + *W*_i_(*k*)) accounts for the fact that wealthier (i.e. more successful) agents will be less prone to consider the opinion of their neighbors. Note that *ζ* = 0 and *ζ* = 1 are representative of the cases of stubborn non-social agents, which neglect the opinions of the others, and of insecure social agents, who tend to disregard their individual predictions in favor of those of their neighbors, respectively.

#### Placing an order

*Determining the theoretical demand*. Following the approach presented in [30], agent *i* decides the quantity to buy/sell by maximizing the the expected value of her utility function, that is, the theoretical demand *π*_*i*_ of agent *i* at time *k* is given by

$$\begin{array}{c} \pi \left(k\right)=\underset{\pi }{arg max}E\left[U^{i}\left(W_{i}\left(k,\pi \right)\right)\right], \end{array}$$ (6)

where *W*_*i*_(*k*, *π*) = *c*(*k*) + *πp*(*k*). Two of the main classes of utility functions typically employed to model the agent behaviors are the CARA (constant absolute risk aversion) and CRRA (constant relative risk aversion) functions [32], which are defined as

$$\begin{array}{c} U_{\text{CARA}}^{i}(W_{i}\left(k,\pi \right),\alpha_{i})=-e^{-\alpha_{i}W_{i}\left(k,\pi \right)} \end{array}$$ (7)

and

$$\begin{array}{c} U_{\text{CRRA}}^{i}(W_{i}\left(k,\pi \right),\varsigma_{i})=(W_{i}{\left(k,\pi \right)}_{i}^{\varsigma }-1)/\varsigma_{i}, \end{array}$$ (8)

respectively; the quantities *α*_*i*_ and 1 − *ς*_*i*_ represent the risk aversion of the *i*-th agent. In the absence of dividends and risk-free assets, an approximate solution of (6) is

$$\begin{array}{c} \pi_{i}\left(k\right)=\frac{{\hat{r}}_{i}\left(k\right)}{\varrho_{i}{\hat{V}}_{i}\left(k\right)p_{\text{cur}}^{i}\left(k\right)}, \end{array}$$ (9)

where the risk aversion *ϱ*_*i*_ is equal to *α*_*i*_ if a CARA utility function is selected, while it is 1 − *ς*_*i*_ if a CRRA utility function is used instead, see the Appendixes of [30] and [33] for the formal derivations; ${\hat{V}}_{i}\left(k\right)$ is the estimated volatility at the *k*-th day of trading, computed from the returns of the asset in the past *τ*_*i*_ trading sessions, that is,

$$\begin{array}{c} {\hat{V}}_{i}\left(k\right)=\frac{1}{\tau_{i}}\sum_{\delta =1}^{\tau_{i}}{\left(r\left(k-\delta \right)-{\bar{r}}_{i}\left(k\right)\right)}^{2}, \end{array}$$ (10)

where *r*(*k*) = ln(*p*(*k*)/*p*(*k* − 1)) is the spot return of the asset price at day *k* − *i*, while ${\bar{r}}_{i}\left(k\right)$ is the average spot return in the trading sessions *k* − 1, …, *k* − *τ*_*i*_. The theoretical demand *π*_*i*_(*k*) is then compared with the quantity *a*_*i*_(*k*) that the agent currently possesses to determine whether a buy or sell order has to be submitted. Note that, as in [30, 33], an agent can neither leverage debt to buy more shares, nor short sale, that is, she can only sell shares of the asset she currently owns.

*Price determination*. Similar to [34], the price set by the *i*-th agent is distributed around the bid (in case of a buy order) or ask price (in case of a sell order). Namely,

$$\begin{array}{c} p_{i}\left(k\right)=\{\begin{array}{ccc} p_{\text{bid}}^{i}\left(k\right)-\left(\eta_{i}\left(k\right)-m\right), & \text{if}\;\pi_{i}\left(k\right)>a_{i}\left(k\right) & \text{(buy}\;\text{order)},\;\\ p_{\text{ask}}^{i}\left(k\right)+\left(\eta_{i}\left(k\right)-m\right), & \text{if}\;\pi_{i}\left(k\right)<a_{i}\left(k\right) & \text{(sell}\;\text{order)}, \end{array} \end{array}$$ (11)

where $p_{\text{bid}}^{i}\left(k\right)$ and $p_{\text{ask}}^{i}\left(k\right)$ are the bid and ask price before agent *i* participates to the *k*-th trading session, and *η*_*i*_(*k*) is the realization of a log-normal random variable with meta-parameters defined as in [34], that is, with mean 7 and standard deviation 10; the shift parameter *m* is the median of the distribution, and regulates the tradeoff between market and limit orders. The log-normal distribution is selected in accordance with the empirical observations on the order distribution in the book: counter-intuitively, submitted orders are not always market orders or limit orders slightly above the best quote, but may also strongly deviate from $p_{\text{bid}}^{i}$ (or $p_{\text{ask}}^{i}$) [35]. This behaviour has been explained in line with the trading strategies adopted by some traders, as for instance the *stop loss* The strategy consists in submitting an order in the opposite direction of the current position to cap the maximum possible loss in case of a sudden adverse price variation.

*Determining the order size*. Agent *i* then compares the theoretical demand *π*_*i*_(*k*) with the current shares *a*_*i*_(*k*) she possesses, and with her current resource availability *c*_*i*_(*k*), thus deriving the order size *d*_*i*_(*k*) as

$$\begin{array}{c} d_{i}\left(k\right)=\{\begin{array}{ccc} \left\lfloor \min(\pi_{i}\left(k\right)-a_{i}\left(k\right),c_{i}\left(k\right)/p_{i}\left(k\right))\right\rfloor & \text{if}\;\pi_{i}\left(k\right)>a_{i}\left(k\right) & \text{(buy}\;\text{order),}\;\\ \left\lfloor \min(a_{i}\left(k\right)-\pi_{i}\left(k\right),a_{i}\left(k\right))\right\rfloor & \text{if}\;\pi_{i}\left(k\right)<a_{i}\left(k\right) & \text{(sell}\;\text{order),} \end{array} \end{array}$$ (12)

where ⌊⋅⌋ is the floor function. Subsequently, agent *i* places a buy (or sell) order of *d*_*i*_(*k*) units of the asset at price *p*_*i*_(*k*), which is registered in the book.

### Measuring herding and efficiency

To adequately quantify the intuitive concepts of herding and market efficiency, we introduce the following metrics:

- denoting ${\hat{p}}_{i}\left(k\right)=p_{\text{cur}}^{i}\left(k\right)\exp\left({\hat{r}}_{i}\left(k\right)\right)$ the price estimation performed by agent *i*, and [*t*_0_, *T*] the window of trading under analysis, we define the percent **herding intensity** *H* as
  $$H=100\frac{{\left\|\rho \right\|}_{2}-1}{N-1},$$
  where *ρ* is the sample correlation matrix of the time series ${\hat{p}}_{i}\left(t_{0}\right),\ldots ,{\hat{p}}_{i}\left(T\right)$ of all agents *i* = 1, …, *N*, whose element *lm* is
  $$\rho_{lm}=\frac{\sigma_{lm}^{2}}{\sigma_{ll}\sigma_{mm}},$$
  with
  $$\sigma_{lm}=\frac{1}{T-1}{\left(\sum_{t=t_{o}}^{T}\left({\hat{p}}_{l}(k)-\langle {\hat{p}}_{l}\rangle )({\hat{p}}_{m}(k)-\langle {\hat{p}}_{m}\rangle \right)\right)}^{1/2},$$
  and $\langle {\hat{p}}_{i}\rangle$ being the average ${\hat{p}}_{i}$ in the trading window under analysis. Note that 0 ≤ *H* ≤ 100, with *H* = 0 in the absence of correlation, and *H* = 100 when all the agents perform the same estimation throughout the trading window, that is, when ${\hat{p}}_{i}\left(k\right)={\hat{p}}_{j}\left(k\right)$ for all (*i*, *j*) and for all *k* ∈ [*t*_0_, *T*].
- introducing the error *e*(*k*) ≔ *p*(*k*) − *p*_*f*_(*k*) between the market and fundamental prices, we define the **market inefficiency** $\bar{e}$ as
  $$\bar{e}=\frac{1}{T}\sqrt{\sum_{t=t_{o}}^{T}e{\left(k\right)}^{2}}.$$

## Results

Here, we numerically investigate the properties of the model presented above to elucidate the relationship between sociability, herding, and market efficiency. The outcome of this analysis is then leveraged to propose market policies that enhance its efficiency. Finally, we evaluate how variations in the maximum risk aversion of the traders impact on market dynamics.

### Numerical setup

We consider an artificial financial market composed of *N* = 100 agents, whose pattern of interactions is captured by a Barabasi-Albert scale-free network [36] with average degree 4. A scale-free network topology has been selected as it is typically observed both in financial networks and in other social or biological contexts [37–40]. In agreement with past works, the fundamental price *p*_*f*_(*t*) is taken as a realization of a geometric Wiener process [30, 31]. The agents’ initial capital, wealth and return expectation, as well as the random distributions from which the model parameters are extracted, are reported in Table 1, and each simulation considers a time frame of *T* = 1000 trading sessions. Since the order book is empty at the beginning of each simulation, we consider $p_{\text{cur}}^{i}\left(0\right)=p_{\text{bid}}^{i}\left(0\right)=p_{\text{ask}}^{i}\left(0\right)=p_{f}\left(0\right)$, and we start computing herding intensity and market efficiency from *t*_0_ = ⌊3*T*/10⌋. In any trading session in which no buy (sell) orders are present in the book, both $p_{\text{bid}}^{i}$ ($p_{\text{ask}}^{i}$) and $p_{\text{cur}}^{i}$ are set to $p_{\text{ask}}^{i}$ ($p_{\text{bid}}^{i}$). As wealth is considered as a proxy of success, the parameters have been set so that the wealthiest node is also the most influential, that is, the node, say *i*, with the highest out-degree $d_{i}^{\text{out}}$, defined as the the cardinality of the out-neighbors set

$$N_{i}^{\text{out}}=\left\{i\in V:\left(i,j\right)\in E\right\}.$$

**Table 1:** Market initialization and selected distribution for each of the parameters.

Variable	Initial value	Parameter	Distribution
*c*_*i*_(*k*)	$3000{\left(1+d_{i}^{\text{out}}\right)}^{2}$	*ϱ*_*i*_	U(0,0.005)
*a*_*i*_(*k*)	100	*γ*_*i*_	$N(0.5,0.1^{2})$
${\hat{r}}_{i}\left(k\right)$	0	*τ*_*i*_	$N(50,10^{2})$
		*σ*_*i*_	N(5,1)

To test for significant variations of the metrics *H* and $\bar{e}$ depending on the level of sociality or on the adopted market policies, one-way ANOVAs are employed in the following analyses. In particular, when the data in the group are non-normal (normality is verified with a Lilliefors test [41]) or when the two groups are heteroskedastic (different variance between the two groups, verified with the Bartlett test [42]), suitable transformations of the data have been applied before performing the ANOVA test.

### Sociability, herding, and market behavior

**Fig 1:**
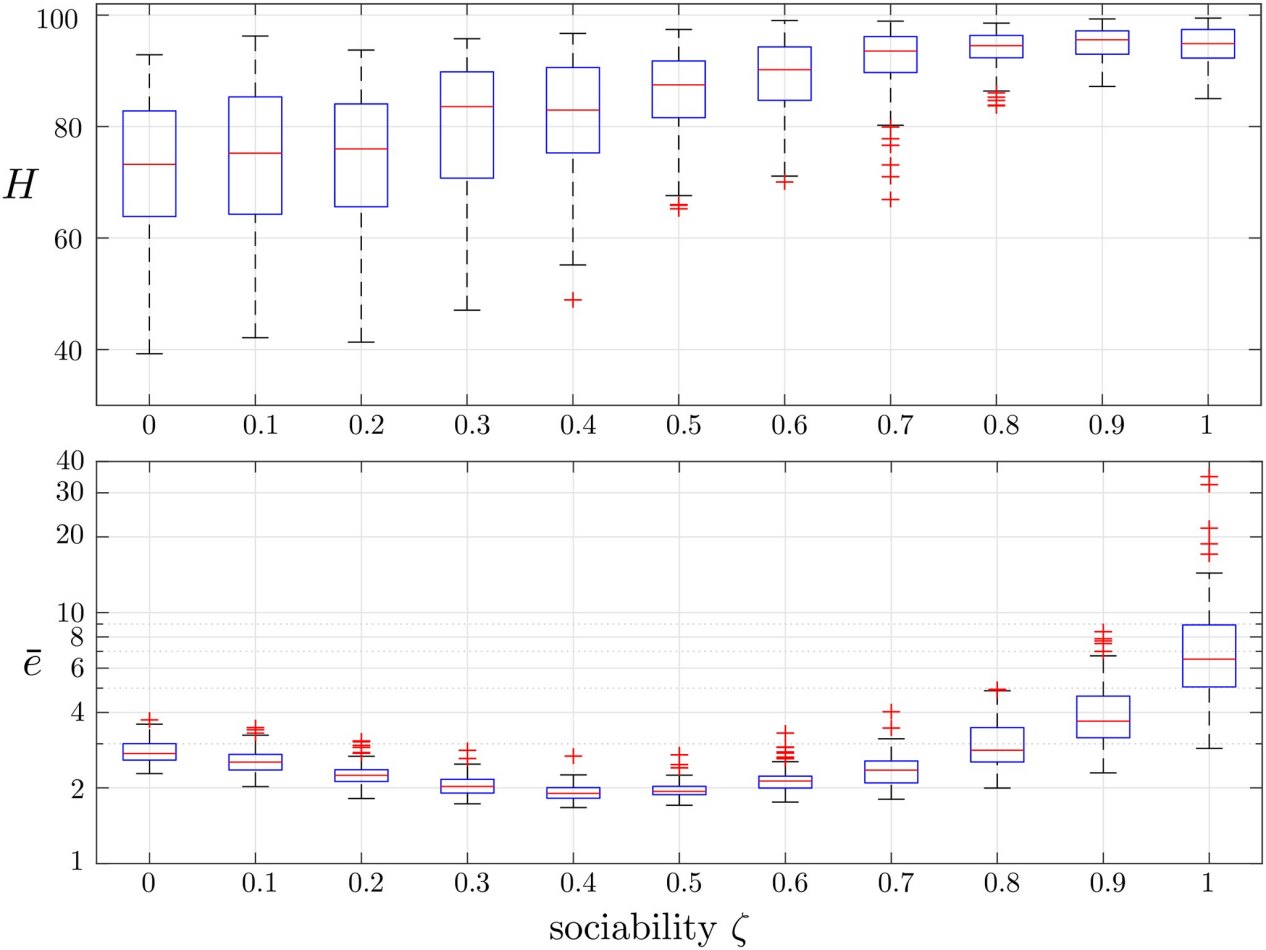
Box plots of the realizations of the herding intensity *H* (top panel) and the market efficiency $\bar{e}$ (bottom panel) obtained with 100 simulations for each value of *ζ*.

To investigate how the mutual influence among the investors affects the market dynamics, we vary the sociability *ζ* in the set [0, 1] with step 0.1 and, for each value of *ζ*, run 100 simulations to assess the statistical relevance of the observed results. For each value of *ζ* ≠ 0, we performed a one-way ANOVA to test whether herding intensity and efficiency were different from the case *ζ* = 0, where sociability is absent, and we always obtained statistical significance, with *p*-values smaller then 0.005 (except for herding when *ζ* ≤ 0.2). In Fig 1, we report the box-plots of *H* and $\bar{e}$ as a function of the sociability parameter *ζ*. We notice that, while the herding intensity monotonically increases with the sociability, its impact on $\bar{e}$ is less trivial. Indeed, an increment in *ζ* is initially beneficial for the efficiency, while when *ζ* ≥ 0.8 market efficiency dramatically drops.

**Fig 2:**
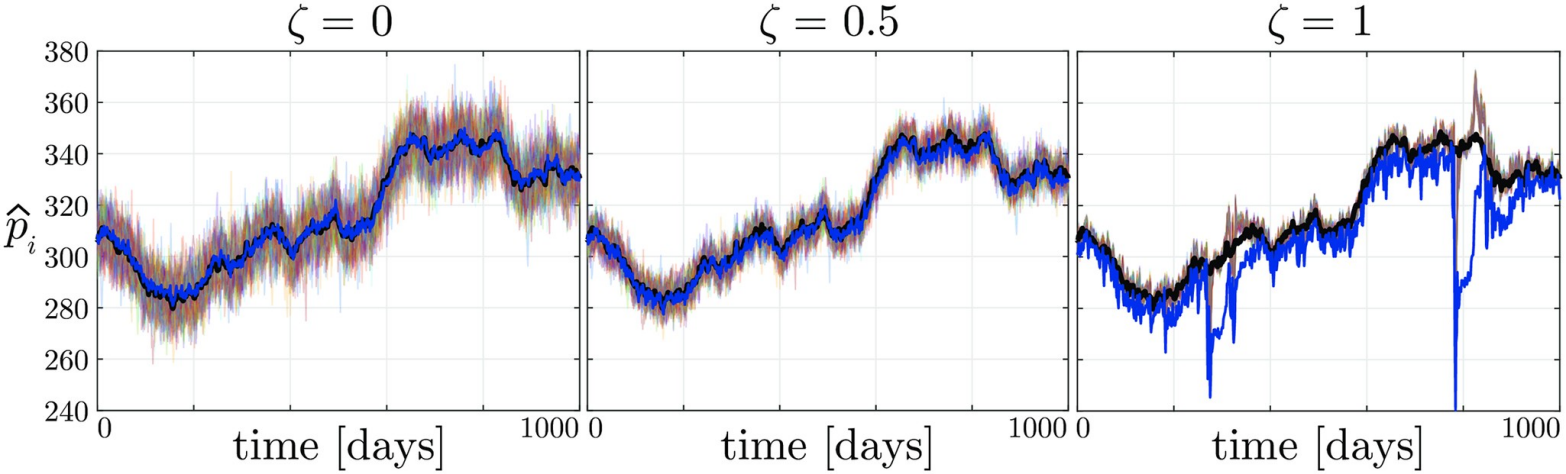
Dynamics of the asset price estimates performed by the traders for three sample simulations with the same fundamental price *p*_*f*_(*k*) (black line), and only differing in the sociability *ζ*. The dynamics of the market price *p*(*k*) are depicted in blue.

To further illustrate the relationship between herding and efficiency, we report in Fig 2 the time series of the expected prices ${\hat{p}}_{i}$ of three sample simulations of our model with *ζ* = 0, 0.5, and 1, respectively. In the absence of sociability (Fig 2, left panel) the market is apparently efficient, since the market price *p*(*k*)–blue line in the figure–tracks the fundamental price *p*_*f*_(*k*). This is the result of the fact that our double-auction market, when determining the market price, tends to average the price expectations of the different agents, which range around *p*_*f*_(*t*) in a band of width 20. Note that increasing *ζ* has a twofold effect on the market dynamics. Indeed, it both reduces the variance of the expected fundamental price ${\hat{p}}_{i}$ across the agents, and steers the ${\hat{p}}_{i}$-s towards the expectation of the most influential (wealthy) nodes. For moderately high values of *ζ* (see the central panel of Fig 2), this improves market efficiency, moving the market price closer to the fundamental price, while for higher values of *ζ* (Fig 2, right panel), this can produces strong deviations of *p*(*k*) from *p*_*f*_(*k*), thus generating market bubbles (where the market price differs more than 20% from the fundamental price) and dramatically reducing efficiency.

### Controlling market efficiency

The analyses performed in the previous section illustrates how an increase of the herding intensity is not necessarily detrimental for market efficiency. However, when all the agents tend to conform their expectations to those of a group of influential leaders, efficiency is lost and market bubbles may emerge. In social trading, the market platform (as, for instance, *eToro* and *Zulutrade* [15]) provides the infrastructure to display profiles of the traders to other users. Therefore, the platform can manipulate the topology of interconnection and the kind of information shared among the investors. In what follows, we propose and numerically validate two alternative control strategies to enhance market efficiency, which can be translated into implementable market policies. Both strategies aim at avoiding that the opinion of a few determined the expectation of the group. The first control strategy, denoted *isolating the hubs*, prescribes that the strategies of the most influencial nodes are not spread through the platform, while the second approach, denoted *anonymization*, consists in not disclosing the identity of the agents across the network. To test the effectiveness of the two policies, in the following we refer to the same numerical setup used to test the properties of the model in the absence of control.

#### Isolating the hubs

This control strategy leverages the vulnerability of scale-free networks to targeted attacks, which may disconnect the network by only removing the 2% of the most connected nodes [43]. Indeed, our market policy consists in selecting a subset composed of the most connected (the wealthiest) agents in the network, identified in the network as the nodes with the highest out-degree, and neutralize their influence by removing all their outgoing edges.

**Fig 3:**
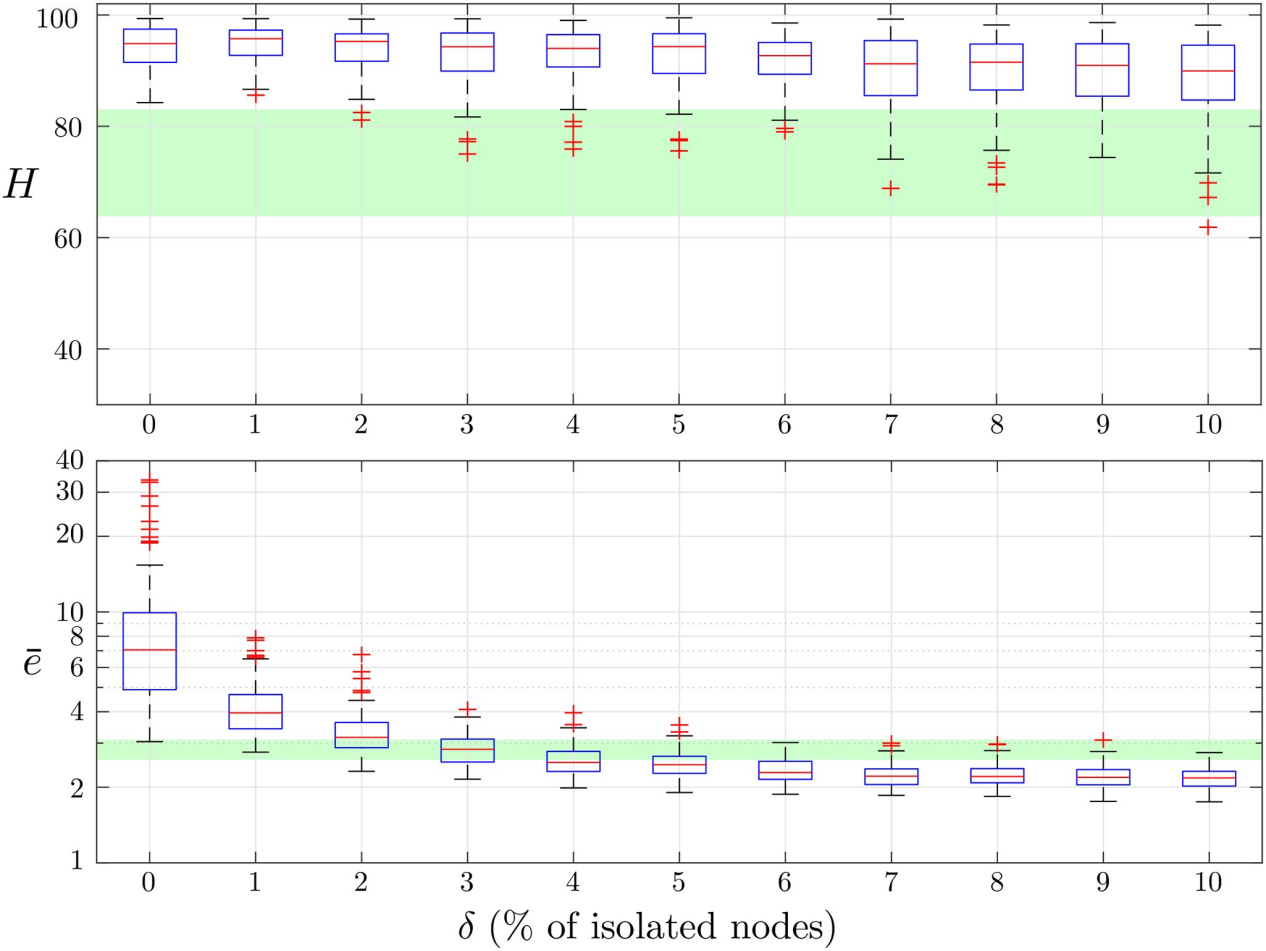
Box plots of the realizations of the herding intensity *H* (top panel) and the market efficiency $\bar{e}$ (bottom panel) with 100 simulations for each value of *δ*. The green-shaded area corresponds to the box obtained for the case *ζ* = 0.

To assess the effectiveness of this approach, we focus on the case in which the uncontrolled market is less efficient, that is, when *ζ* = 1. Then, we start to control (i.e. isolate) an increasing percentage *δ* (from 1 to 10, with step 1) of the most connected nodes. From Fig 3, we notice that, as the number of isolated nodes increases, the market regains efficiency. For each value of *δ*, 100 simulations were run, and a one-way ANOVA has been performed to test whether the herding intensity and market efficiency were significantly different from the case *ζ* = 0, and we always obtained a *p*-value smaller then 0.005 (except the market inefficiency when the 3% of the nodes are isolated). We observe that, as *δ* increases, while the herding intensity remains substantially higher than when sociability is naught, market inefficiency becomes smaller than the case *ζ* = 0 for *δ* > 5 (see the green bands in Fig 3), further supporting the thesis that herding is not necessarily detrimental.

#### Anonymization

**Fig 4:**
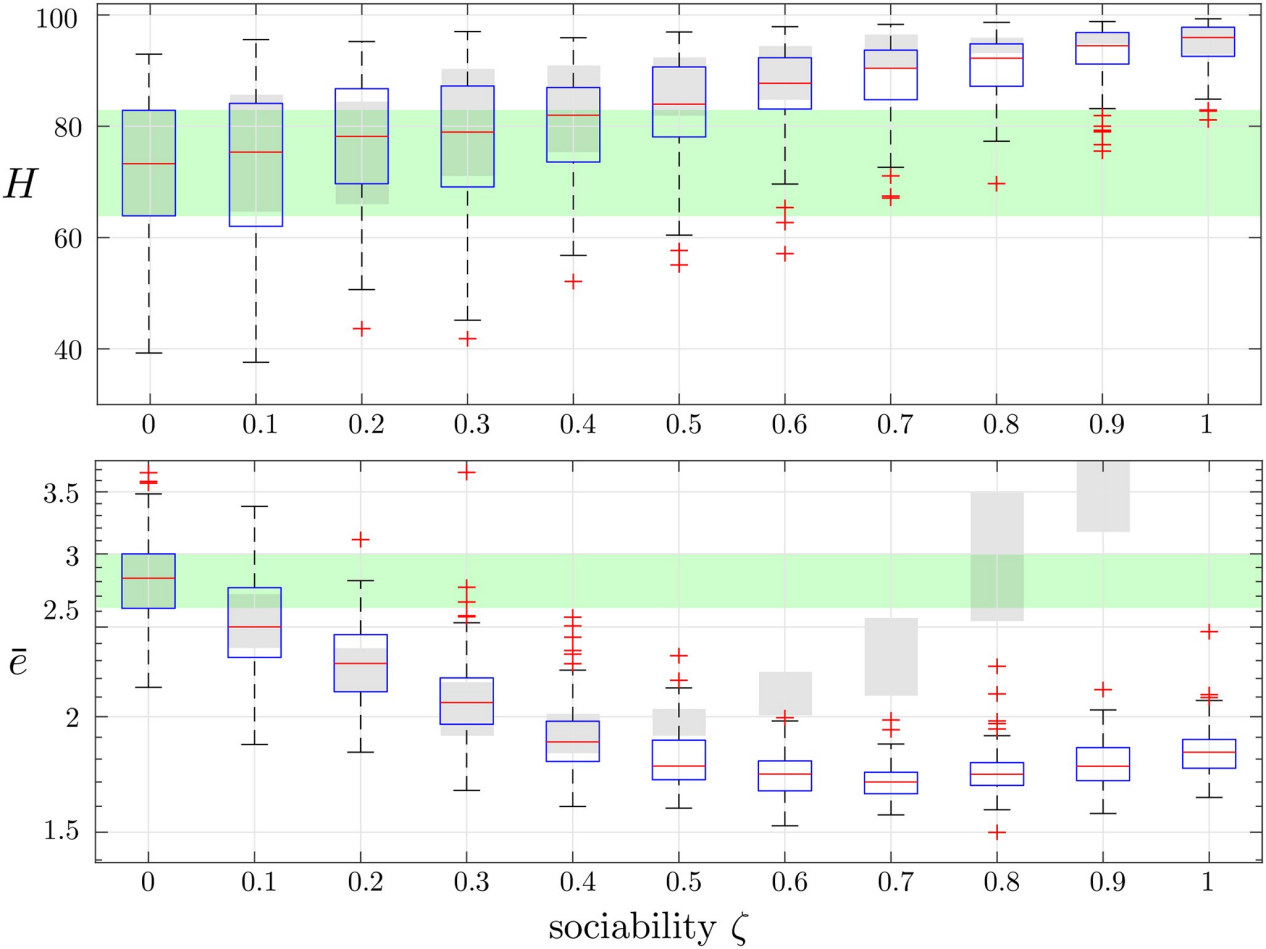
Box plots of the realizations of the herding intensity *H* (top panel) and the market efficiency $\bar{e}$ (bottom panel) obtained with 100 simulations of our model for each value of *ζ* when data are anonymized. The green-shaded area corresponds to the box obtained for the case *ζ* = 0. The gray-shaded boxes replicate those reported in Fig 1 in the absence of anonymization.

The second control action we propose is to anonymize the data exchanged among the traders. More specifically, each investor will receive from her neighbors only the expected return of the traded asset, but will not be aware of her identity. Therefore, she will weight equally the opinions of agents with heterogeneous wealth. When sociability is high, this mechanism will avoid the pathological condition in which all the agents follow the expectation of a single trader, but they will rather follow the *wisdom of the crowd* and the market price will tend to converge towards the average of the individual price estimation, which can be considered as a good approximation of the fundamental price. As illustrated in Fig 4, for all values of sociability, the anonymization strategy only mildly affects the herding intensity (see the comparison with the grey boxes in the top-panel). On the other hand, the anonymization policy makes sociality always beneficial for market efficiency. Indeed, for all *ζ* ≠ 0, the average inefficiency $\bar{e}$ is always reduced if compared with the case of absence of sociality (see the green band in the bottom panel), and this difference is statistically significant for all *ζ* > 0.2 (one-way ANOVAs, *p* < 10^−4^).

By comparing the box plots in the absence and in the presence of the anonymization strategy (see the comparison with the grey boxes in the bottom panel of Fig 4), we observe that, for low values of the sociability, the choice of not disclosing the wealth has a negligible impact of market efficiency. Indeed, the reduced level of sociality avoids the prevalence of opinions of one agent on the rest of the traders even when the wealth is disclosed. However, when *ζ* ≥ 0.5, anonymizing the data becomes crucial to preserve market efficiency, and a one-way ANOVA comparing the market with and without anonymization confirms a significant difference (*p* < 10^−4^).

### Robustness to variations in the risk aversion

Here, we evaluate the robustness of our findings to variations in the risk aversion of the traders. Specifically, we evaluated the effect of sociality for 10 selected values of the maximum risk attitude *ϱ*_max_, equally spaced on a logarithmic scale between 10^−3^ and 0.1 (*ϱ*_max_ was set to 5 × 10^−3^ in Table 1). Values of *ϱ*_max_ beyond 0.1 are not considered since they would correspond to excessively risk averse agents, thus yielding a market where no-one is willing to buy.

**Fig 5:**
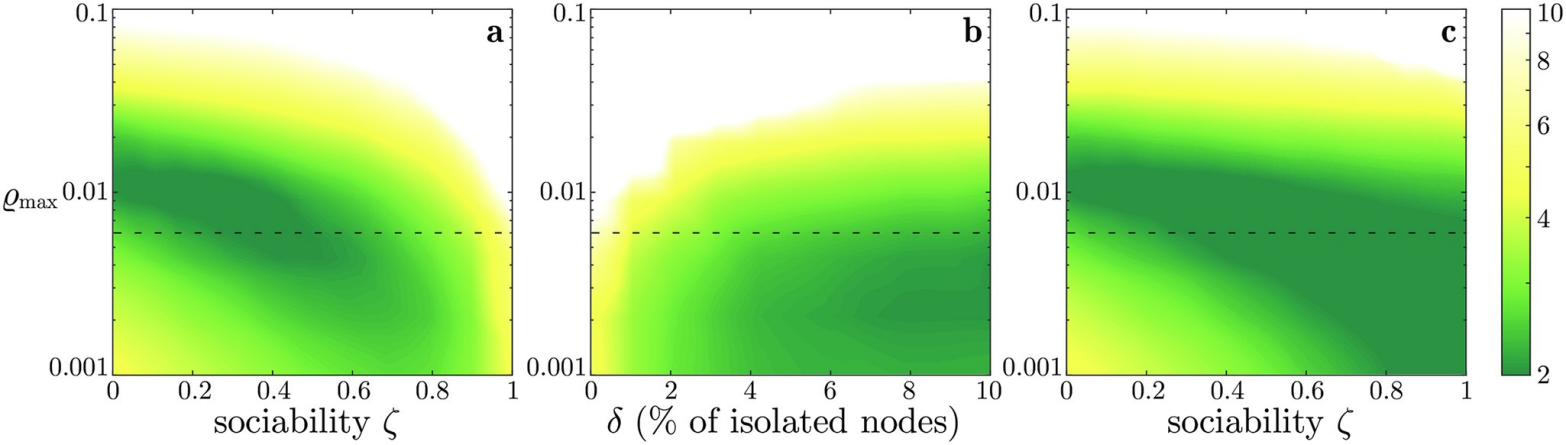
In all panels, the colorbar corresponds to the median $\tilde{e}$ of the market inefficiency $\bar{e}$, obtained from 100 simulations for each parameter combinations. Panels a and c depicts $\tilde{e}$ as a function of the sociability *ζ* and of the maximal risk aversion *ϱ*_max_ with or without anonymization, respectively, while Panel b refers to the strategy *isolating the hubs* and depicts $\tilde{e}$ as a function of the percentage *δ* of isolated nodes and of *ϱ*_max_. The dashed horizontal line corresponds to the value *ϱ*_max_ selected in Table 1.

We notice that, in the absence of a policy to improve market efficiency, when the agents conform to the opinion of the most influential traders, market efficiency worsens, independent of the risk aversion of the agents, see Fig 5a. For all *ϱ*_max_ smaller than 0.01, we identify a tipping point in the sociability *ζ* where we observe the transition from crowd wisdom to herding. The presence of a tipping point from beneficial to detrimental sociality is consistent with the findings in [44], where the authors highlight that this transition happens when the opinion of the agents conforms to that of a group of a few *experts*, which in our work would correspond to the wealthiest (i.e., most successful) traders. When *ϱ*_max_ increases, the tipping point becomes smaller and smaller, and, when the agents are highly averse toward risk (*ϱ*_max_ > 0.01), sociality can only increase inefficiency.

The effectiveness of the proposed policies to limit market inefficiency is confirmed both for the *isolating the hubs* and the *anonymization* strategy, independent of the risk aversion. Indeed, a comparison of Panels a and c in Fig 5 shows how the median market efficiency is always higher when the identity of the traders is not disclosed. Similarly, increasing the percentage *δ* of isolated hubs always yields an increase in market efficiency, see Fig 5b. Note also that the proposed policies also reduce the prevalence of the white color in all panels, which corresponds to a market where no trades occur, since the agents are too averse to risk.

## Discussion

Leveraging a recently proposed artificial double-auction market, the analyses performed in this manuscript contribute a possible solution of the apparent dichotomy between herding and wisdom of the crowd. Indeed, although the presence of imitative behaviors in financial market is indisputable, contrasting empirical observations suggested that they can both be beneficial (hence called *crowd wisdom* or *rational adaptation*) or detrimental (hence called *herding*) for the market efficiency. Our numerical analyses illustrate that, when social interaction among the agents is present but not predominant, imitative behaviors can be beneficial. Indeed, each agent still considers her own estimation of fundamental price *p*_*f*_, but, combining it with the opinion of her neighbors, the variance of her price expectations shrinks, thus reducing the fluctuations around *p*_*f*_. When social expectation dominates the individual estimations, the price expectations of all the traders will tend to coincide. However, since the agent will weight differently the neighbors’ opinion depending on their wealth, their expectations will conform to those of few wealthier agents, similarly to what is observed in weighted and/or signed consensus protocols in opinion dynamics [45–47]. As a result, when the wealthier agents misestimate the fundamental price, the other agents blindly follow, and market bubbles may emerge. In a different context, a similar effect of sociality has been illustrated in the study by Lorenz *et al*. [48], where a group of participants had to solve different estimation tasks about geographical facts and crime statistics. The authors showed that the estimation accuracy can be undermined by sociality. As in our financial market model, a moderate advantage may only persist when sociality is modest, while the accuracy of the estimation substantially worsens when the crowd follows the opinion of a few highly confident individuals.

Considering the impact that sociality has on price estimation, we developed two alternative strategies to contain market inefficiency also when the social interaction among the agents is prevalent. In particular, we assumed that the market institution can manipulate the network topology in order to prevent instabilities. This is the case, for instance, of social trading platforms that may decide the kind of information shared among the agent, and to appropriately tailor the interaction topology. The first proposed control strategy aims at limiting the influence of a small fraction of the network nodes by eliminating their outgoing edges. Translated into a policy for the social trading platforms, this consists in not disclosing the trading strategies of the wealthiest agents. Our results showed that it suffices to adopt this policy on the wealthiest 3% of the traders to avoid the prevalence of the expectations of a few on the market sentiment, thus regaining efficiency. A second alternative control strategy consists instead in anonymizing the expectations exchanged among the agents. This means that the platform forwards to the out-neighbors of a node only her expectation, but not her wealth. In this way, every agent is going to equally account for the expectations of all her neighbors. The efficacy of this egalitarian mechanism is that it filters the most extreme expectations. In this way, the effect of sociality is solely beneficial, since it both reduces the variance of the price expectations, and centers their mean on the fundamental price. There are several directions along which our research can be extended. Since previous work suggested that herding may be more prominent in a multi-asset market [49], the efficacy of the proposed strategy in containing market inefficiency should also be tested when one or more assets are added in the market. Furthermore, experimental observations from world-wide financial markets and recent numerical studies have reported the possible emergence of multiple clusters of herding [50, 51]. Extending the model at a larger scale could be instrumental to identifying how the behavioral and topological features of the market may combine to determine clustered trading patterns.
